# Case Report: Toxic megacolon secondary to chronic constipation and cocaine consumption

**DOI:** 10.3389/fsurg.2024.1434523

**Published:** 2024-08-21

**Authors:** Bertha Dimas, Guillermo Hernández, Ivonne Peralta, Ansony Godinez, Gabriela Gutierrez, Fernando Cruz

**Affiliations:** ^1^ Surgery Unit, Hospital General de México (HGM), Mexico City, Mexico; ^2^ Anesthesiology Unit, Centro Médico ABC, Mexico City, Mexico

**Keywords:** toxic megacolon, chronic constipation, cocaine abuse, bowel perforation, total colectomy

## Abstract

Toxic megacolon (TM) is a severe condition characterized by acute colonic dilation, with specific radiological and clinical signs. The multifactorial etiology of TM is primarily associated with inflammatory bowel disease and infections. However, TM remains a challenging complication due to its potential for rapid progression to life-threatening conditions. This report describes a rare case of TM in a 25-year-old male with a history of recurrent constipation and chronic cocaine consumption. Examination and imaging indicated acute intestinal obstruction with dilated colon segments and fecal impaction, necessitating an urgent laparotomy. Surgery revealed pan-colonic dilatation and sigmoid perforations, leading to a total colectomy and ileostomy. Chronic constipation, often perceived as benign, can escalate into a critical situation, possibly exacerbated by cocaine-induced muscle weakness and hypoxia. Evidence suggests that cocaine negatively affects the intestinal mucosa, potentially leading to ischemia. Chronic factors, including the use of enemas, may have contributed to megacolon development and perforation. Overall, this report underscores the critical elements of diagnosis and the importance of patients’ medical history, particularly those with unusual risk profiles. In addition, it highlights the need for further research to fully understand the implications of these cases.

## 1 Introduction

Toxic megacolon (TM) represents a critical medical condition characterized by acute colonic dilation. Radiologically, colonic distension exceeding 6 cm in diameter also presents with clinical and laboratory signs indicative of systemic toxicity (1). Although rare, this severe complication continues to pose significant challenges, primarily because of its potential for rapid progression to life-threatening conditions (2). In defiance of advances in medical knowledge and treatment modalities, the intrahospital mortality rate associated with TM remains unsatisfactory at approximately 7.9% (3). The pathogenesis of TM is multifactorial, with associations primarily linked to inflammatory bowel diseases, particularly ulcerative colitis and infections that lead to colonic dysmotility and dilation (2, 4). Furthermore, TM can result from chronic conditions that predispose individuals to colonic inertia and subsequent distension, among which chronic constipation and cocaine use stand out as less common, but plausible etiological factors.

The use of cocaine may have various adverse effects on the gastrointestinal system; however, a direct association with toxic megacolon has not yet been established. Although no specific study has directly linked cocaine use to TM, evidence has shown that cocaine use can negatively impact the intestinal mucosa (5). Intestinal ischemia, perforation, bowel infarction, transient neurogenic dysfunction, and necrosis are manifestations of cocaine use (6–9).

Chronic constipation (CC), often managed as a benign and predominantly lifestyle-related condition, can evolve into a critical situation in rare instances, necessitating urgent and aggressive intervention (10, 11). Little is known about the association between cocaine abuse and CC; however, muscular weakness, tissue-level hypoxia, and consequently, muscular atrophy have been described as common factors in both conditions (6, 9, 12). All the aforementioned features of cocaine abuse can be related to CC, making this sum of factors a plausible cause of TM. The case presented herein aims to shed light on this rare progression from chronic constipation to TM, emphasizing the critical aspects of timely diagnosis, adequate medical background interrogation, and importance of considering TM in the differential diagnosis of severe abdominal conditions.

## 2 Case presentation

The patient is a 25-year-old male, with medical history of chronic constipation and chronic cocaine consumption since 2019 as the only relevant medical background. He presented to our national referral institution with abdominal pain, anorexia, and worsening constipation without improvement, despite the use of enema and laxatives. The manifestations started three months before, without decrease or alleviation throughout the study period, despite the consumption of multiple unspecified medications. Abdominal physical examination revealed a distended abdomen with muscle resistance, pain upon superficial and deep palpation in the descending colon area, generalized tympanitic to percussion, and bowel sounds present but reduced in frequency to one sound per minute.

**Figure 1:**
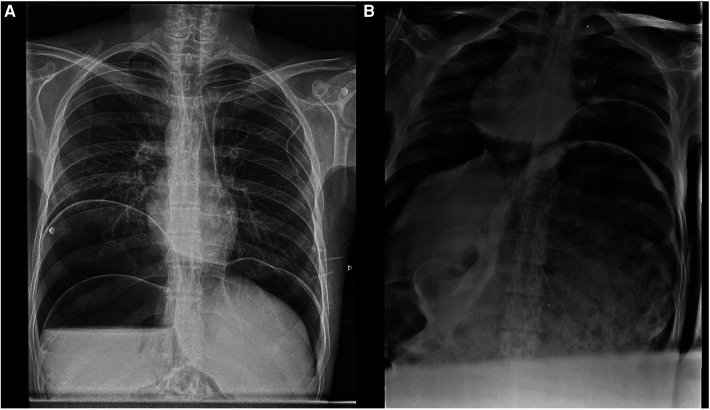
**(A)** Anteroposterior chest radiograph demonstrating significant colonic dilation. The radiograph reveals extensive gaseous distension of the colon, characteristic of toxic megacolon. The diaphragm is elevated, and there is notable colonic distension. **(B)** The image highlights the extent of colonic dilation with air-fluid levels indicative of an obstructive process. The patient exhibits marked gaseous distension of the intestines, consistent with the diagnosis of toxic megacolon.

**Figure 2:**
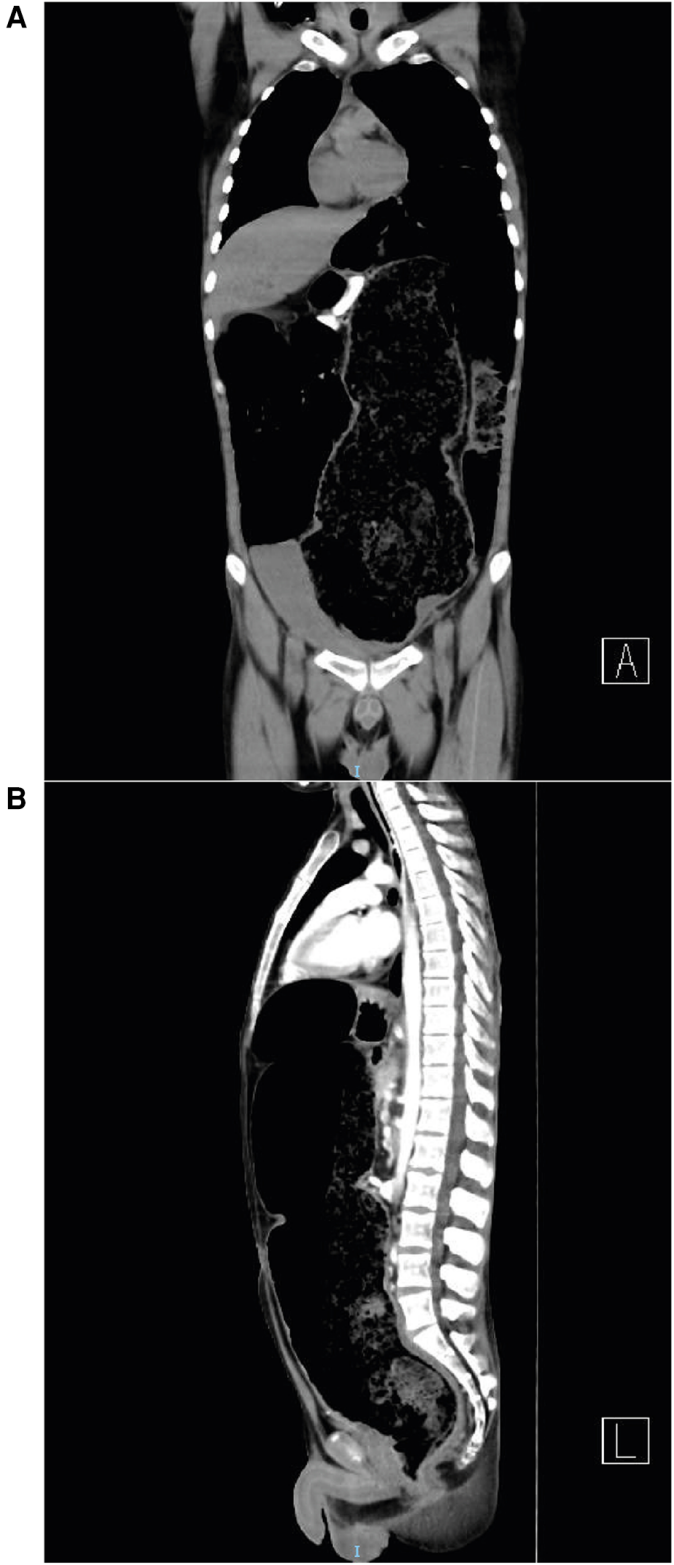
**(A)** Coronal CT scan displaying significant colonic dilation with extensive gaseous distension. **(B)** Sagittal CT scan showing severe abdominal distension and colonic dilation.

Complete blood work was performed, resulting in white cells: 12.9 × 10^3 ^/µl (normal range: 4.5–10.0), neutrophils 79.5% (40–70), hemoglobin 7.1 g/dl (14–18), total platelets 694.00 × 10^3 ^/µl (150–450), creatinine 0.4 mg/dl (0.84–1.25) and lactic acid 1.3 mmol/L (0.5–2.22). Body imaging was then performed. Abdominal anteroposterior supine radiography revealed dilated intestinal loops and air fluid levels, suggesting acute obstruction (Figure 1). Abdominal computed tomography (CT) showed intestinal occlusion due to fecal impaction in the rectum and cecal dilatation of 11.6 cm (Figure 2).

**Figure 3:**
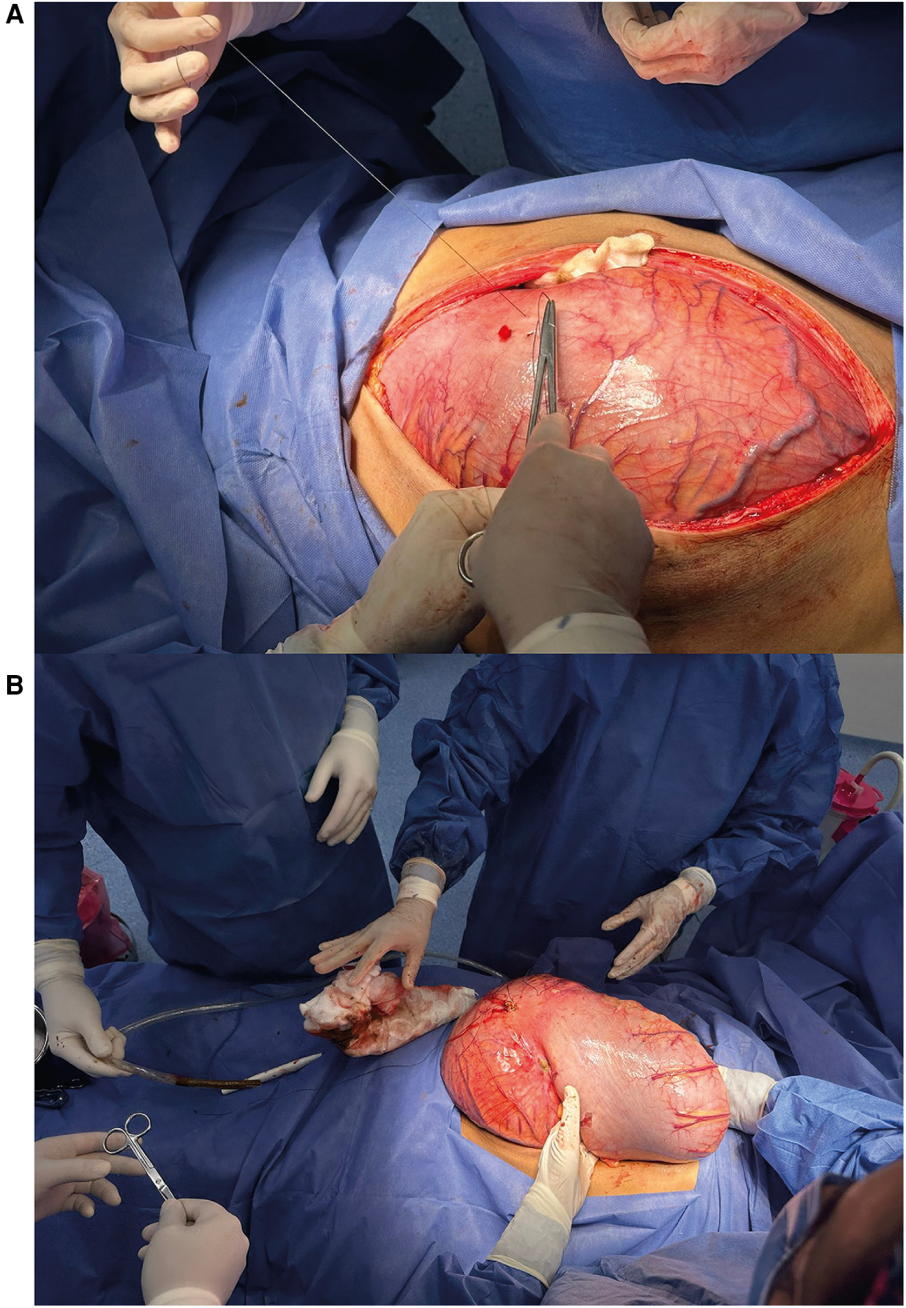
**(A)** Intraoperative view showing the initial incision and exposure of the markedly distended colon. The image demonstrates the surgical approach taken to access the affected bowel. **(B)** Intraoperative image depicting the resected colon during total colectomy. The surgical team is shown handling the excised specimen, which displays severe distension and pathological changes characteristic of toxic megacolon.

Exploratory laparotomy was performed when urgency was identified. Direct colon observation revealed pan-colonic dilatation, with a sigmoid maximum diameter of 15 cm, in the descending colon of 12 cm, and in the ascending and transverse colon of 10 cm. In addition, the patient presented perforations in the anterior (1.5 cm × 1.5 cm) and lateral (1.0 cm × 1.0 cm) portions of sigmoid colon (Figure 3). Total colectomy with ileostomy reconstruction was performed with no complications in the immediate postoperative period.

**Figure 4:**
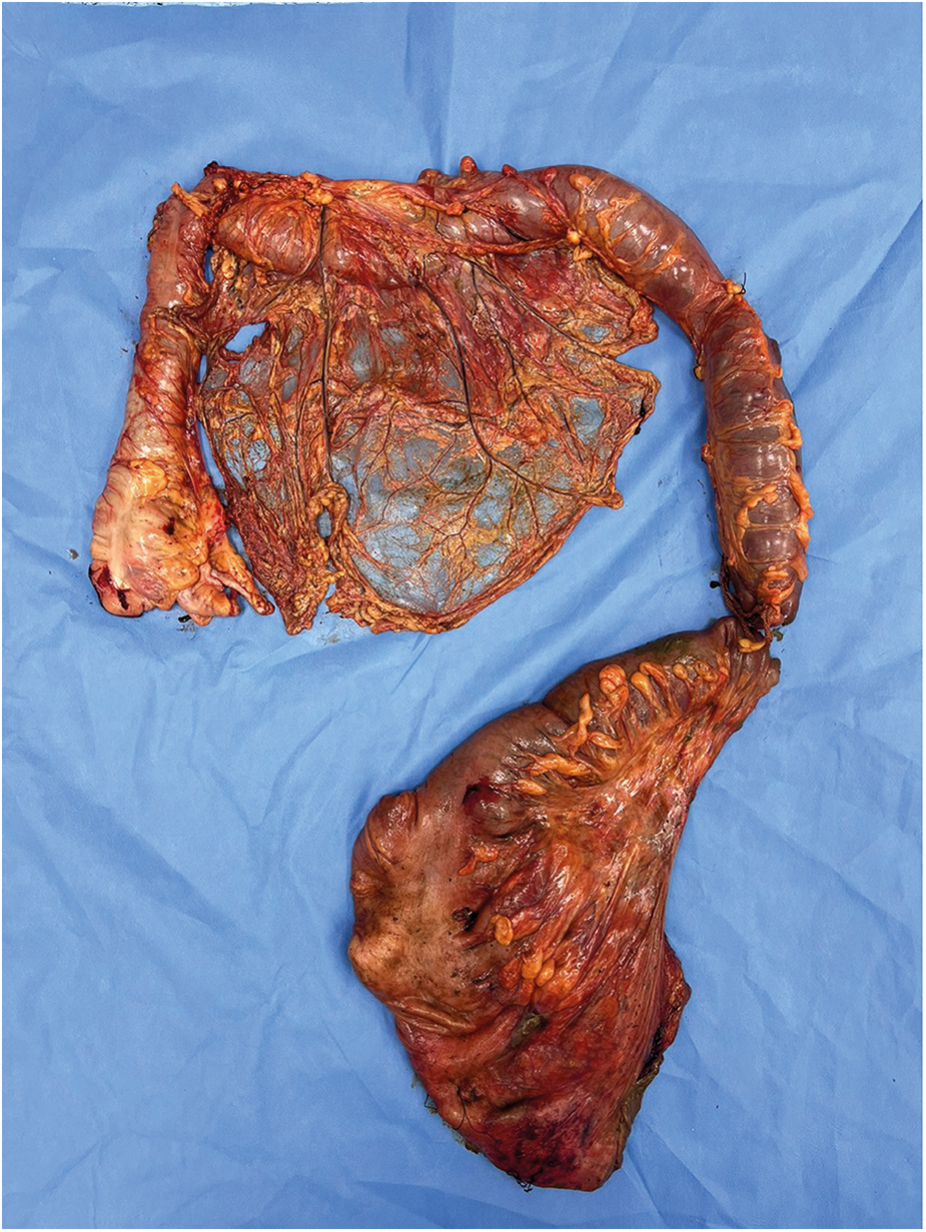
The resected specimen colon is markedly dilated, with extensive areas of congestion, hemorrhage, and ulceration visible on the mucosal surface. The segment exhibits both pan-colonic dilation and structural compromise.

After surgery, the pathological report revealed a congestive, hemorrhagic, and ulcerated appearance of the last 15 cm. Thinned and atrophic walls were also observed. In the sigmoid colon, extensive irregular ulcers were observed on the mucosal surface, measuring up to 20 cm in length and 10 cm in width and deep with raised edges (Figure 4).

On the seventh day of hospitalization, the patient developed signs of systemic immune response with cardiovascular and neurological effects. Therefore, additional intervention was required, in which 1,500 cc of suppurative material was found, demanding abdominal intracavity lavage. Perforation of the urinary system was examined using methylene blue infusion, corroborating the integrity of the urinary bladder and ureters. Leakage signs were observed in the intestinal stump, following a previously described procedure. Once the intestinal slump had been reconstructed without any signs of perforation or leakage, surgery was performed.

After the second intervention, the patient continued to show hemodynamic instability and inadequate neurological response. Unfortunately, the patient died on the eighth day of hospitalization.

## 3 Discussion

In this report, we present a rare case of TM, presumably secondary to chronic constipation and cocaine consumption, in a 25-year-old male. The patient, with a significant history of constipation and cocaine use since 2019, presented with severe abdominal pain, anorexia, and worsening constipation, which was unresponsive to conventional treatment. Despite the surgical intervention, the patient died on the eighth day of hospitalization. This case underscores the criticality of TM as a complication of chronic constipation compounded by cocaine use, and highlights the necessity for timely intervention.

The introduction of toxic megacolon as a severe, life-threatening condition marked by acute colonic dilation and systemic toxicity sets a grim backdrop against which this case unfolds. Although traditionally associated with inflammatory bowel diseases, particularly ulcerative colitis and infections (1, 4), the multifactorial etiology of TM enables the appearance of less common precipitants such as chronic constipation and substance abuse. This case serves to expand our understanding of TM by exploring the role of cocaine, a substance known to adversely affect the gastrointestinal system through mechanisms such as ischemia, perforation, and neurogenic dysfunction, as a contributing factor in the development (6, 9). By situating this case within the broader spectrum of TM pathogenesis, we investigated the critical aspects of diagnosis, importance of a thorough medical history, and need for heightened awareness of TM in differential diagnoses, particularly in patients with atypical risk profiles.

While no direct association has been found between cocaine use, CC, and TM, research indicates that a decrease in intestinal mucosal blood flow and motility are important pathogenic factors for damage to the intestinal mucosal barrier, dysbiosis, and the pathogenesis of low motility and intestinal wall dilatation (13–15). These effects have been observed in several preclinical studies, in which gastrointestinal function and integrity were studied under cocaine use, suggesting mechanisms by which cocaine could impair intestinal health. In addition, cocaine abuse, particularly in combination with alcohol, can induce segmental intestinal ischemia without evident thrombosis in the mesenteric vessels, indicating that vascular lesions and reduced blood flow contribute to intestinal damage (13).

Recently, ghrelin, also known as the “hunger hormone,” has been identified as a modulator of underlying mechanisms in substance use disorders, including those related to cocaine. The ghrelin-GHS1*α*R axis influences homeostatic and hedonic aspects of food intake and mediates reward-related signaling associated with cocaine and opioids (16). Moreover, ghrelin is produced in the gastrointestinal tract and is involved in various functions, including motility and gastric emptying, which are also affected by cocaine use (17).

In addition to the preclinical evidence presented, several case reports underscoring the role of cocaine use in bowel ischemia and perforation have been published. In 2020, a patient with constipation and cocaine consumption who developed chemical colitis secondary to low-dose hydrogen peroxide enema was reported (18). Interestingly, although cocaine and hydrogen peroxide pose risks to bowel integrity, their concurrent use appears to amplify the risk of perforation. The case reported by Galo et al. suggests that even lower doses of hydrogen peroxide, which might otherwise be tolerated, can be dangerous when combined with cocaine abuse. Our experience suggests that concomitant use of hydrogen peroxide and cocaine could lead to dangerous vascular alterations and life-threatening complications. Similarly, our patient had multiple enemas that may have severely affected the disease course. The addition of chemical colitis to the already damaged intestinal mucosa and weakened muscles may have affected the intestinal dilatation and subsequent bowel perforation.

In emergency colorectal surgeries, the risk of postoperative complications, such as surgical site infection (SSI) and subsequent sepsis development, is markedly increased (19). Anastomotic leakage, a frequent complication following colorectal surgery, significantly contributes to the occurrence of postoperative sepsis. The prevalence of these challenges remains considerable. These difficulties carry significant consequences, such as prolonged hospital stays, increased morbidity, readmissions, and high mortality rates (19, 20).

Aseptic techniques, appropriate antibiotic prophylaxis, optimized perioperative management and postoperative monitoring are essential clinical practices. These strategies not only aid in reducing SSI rates but also serve to minimize the financial burden of healthcare and enhance surgical outcomes for colorectal surgery patients (20).

Overall, these studies underscore the importance of considering a wide range of biological and lifestyle factors in the pathogenesis and treatment of gastrointestinal disorders. The relationship between chronic constipation, potential development into more severe conditions such as megacolon, and exacerbating role substances such as cocaine may represent an area for further research, particularly for understanding the full scope of the impact of cocaine on the gut.

Our study highlights the importance of adequate medical history interrogation and the relevance of considering how factors such as substance abuse and the use of self-administered adjuvant treatments affect disease course and outcomes.

## Data Availability

The original contributions presented in the study are included in the article/Supplementary Material, further inquiries can be directed to the corresponding author.
